# Proton Conducting Graphene Oxide/Chitosan Composite Electrolytes as Gate Dielectrics for New-Concept Devices

**DOI:** 10.1038/srep34065

**Published:** 2016-09-30

**Authors:** Ping Feng, Peifu Du, Changjin Wan, Yi Shi, Qing Wan

**Affiliations:** 1 School of Electronic Science and Engineering, and Collaborative Innovation Center of Advanced Microstructures, Nanjing University, Nanjing 210093, China; 2 Key Laboratory of Microelectronic Devices & Integrated Technology, Institute of Microelectronics, Chinese Academy of Sciences, Beijing 100029, China

## Abstract

New-concept devices featuring the characteristics of ultralow operation voltages and low fabrication cost have received increasing attention recently because they can supplement traditional Si-based electronics. Also, organic/inorganic composite systems can offer an attractive strategy to combine the merits of organic and inorganic materials into promising electronic devices. In this report, solution-processed graphene oxide/chitosan composite film was found to be an excellent proton conducting electrolyte with a high specific capacitance of ~3.2 μF/cm^2^ at 1.0 Hz, and it was used to fabricate multi-gate electric double layer transistors. Dual-gate AND logic operation and two-terminal diode operation were realized in a single device. A two-terminal synaptic device was proposed, and some important synaptic behaviors were emulated, which is interesting for neuromorphic systems.

New-concept electronic devices, featuring the characteristics of ultralow operation voltages, low fabrication cost, flexible or wearable etc, have received increasing attention in recent years because they can supplement traditional Si-based electronics that are extensively used in integrated circuits and flat-panel displays^1^,^2^,^3^,^4^,^5^,^6^,^7^,^8^,^9^. To achieve these functions, many cost-effective and eco-friendly materials such as nanomaterials and organic materials as well as simple fabrication methods have been proposed^10^,^11^,^12^,^13^,^14^. For example, to realize ultralow voltage operation, various ionic liquids and electrolytes which have extremely high specific capacitances have been used as gate dielectrics for the fabrication of field-effect transistors^15^. Due to the capacitive electrostatic coupling, an electric double layer (EDL) is formed at the interface between ionic electrolyte and semiconductor channel to neutralize charged interface and the free carrier concentration of the channel can be effectively tuned by a low gate voltage^16^,^17^,^18^. Our group has reported low-voltage oxide-based thin-film transistors based on proton conducting nanogranular SiO_2_ electrolyte films deposited by sol-gel and chemical vapor deposition methods^19^,^20^,^21^. Moreover, artificial synapses, which can implement the cognitive capability of human brain’s neural system with extremely low power consumption, have received much attention as a promising new-concept electronic system^22^,^23^. Presently, memristors and EDL transistors are mainly used as artificial synaptic devices to emulate short-term and long-term synaptic plasticity^24^,^25^,^26^,^27^,^28^,^29^,^30^,^31^. In addition, proton transfer across polymer thin film has been investigated as a new class of biocompatible solid-state devices to control and monitor the flow of protonic current for bioelectronic applications^32^,^33^,^34^,^35^.

On the other hand, organic-inorganic composite systems can offer an attractive strategy to combine the merits of organic and inorganic materials into promising electronic devices and have resulted in the realization of a variety of interesting features^36^,^37^,^38^. Composite materials can extend or provide novel capabilities that are difficult to obtain by using each component individually. For instance, solar cells incorporating semiconductor nanocrystals and conjugated polymers have been demonstrated with the potential to deliver efficient energy conversion with low-cost fabrication^39^. Organic/inorganic hybrid biosensor based on a polymer-coated GaAs device enables high sensitivity to low concentrations of ammonia and long-term protection in harsh environments^40^. Recently, graphene oxide (GO), a related material of graphene, has received much attention benefiting from their respective peculiar structures and properties^41^,^42^,^43^,^44^. GO sheet is an atomically thin sheet of graphite and has oxygen functional groups on their basal planes and edges. It is electronically hybrid featuring both conducting π-states from *sp*^2^ carbon sites and large energy gap between the σ-states of *sp*^*3*^ carbon sites. GO film has very high dielectric constant and could be used as dielectric spacer of superconductors and gate dielectrics of EDL transistors. Chitosan is a naturally abundant, nontoxic cationic biopolymer obtained from deacetylation of chitin and has many applications in biotechnology and biomedicine. Due to its excellent film-forming ability, its applications as the gate dielectric material of transistors have been investigated and good results have been obtained^45^,^46^,^47^,^48^,^49^,^50^.

The performance of transistors critically relies on gate dielectrics, which ideally should have large capacitance for low operation voltage and high robustness for low leakage current, long-term reliability etc. In this work, we demonstrated that solution-processed GO/chitosan composite film was an excellent proton conducting electrolyte film, showing a high specific capacitance of ~3.2 μF/cm^2^ at 1.0 Hz. Such GO/chitosan hybrid material was used as gate dielectrics of self-assembled indium-zinc-oxide (IZO) EDL transistors with multiple lateral gate electrodes. Due to proton-mediated EDL coupling at the dielectric/channel interface, the current across the channel can be effectively tuned. We found that, the bottom gate, the lateral gate and the drain could be used to tune the channel conductance, thereby resulting in the realization of diverse functions of our EDL transistors, such as dual-lateral-gate AND logic and the emulation of synaptic behaviors etc.

## Results and Discussion

**Figure 1:**
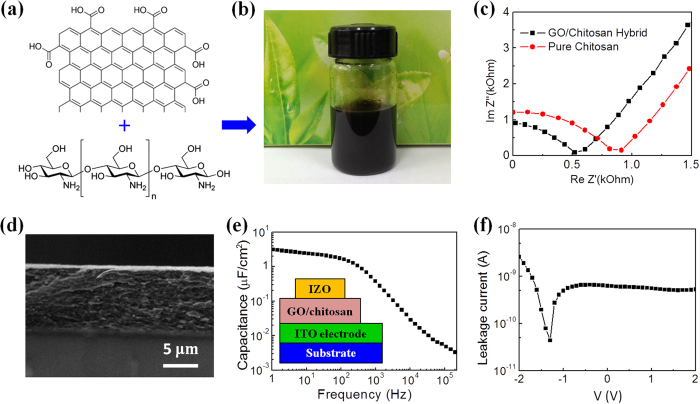
From GO sheets and cihtosan to GO/chitosan composite and the characterization of the composite electrolyte film. (**a**) Chemical structures of GO sheet and chitosan, respectively. (**b**) GO sheets and chitosan were mixed in water solution and stored in a bottle (Taken by P.F.). (**c**) Typical Nyquist plot of the GO/chitosan composite film (black dots). For comparison, Nyquist plot of pure chitosan film is shown (red dots). (**d**) Cross-sectional SEM image of the GO/chitosan composite electrolyte film. The scale bar is 5 μm. (**e**) Specific capacitance of GO/chitosan composite electrolyte film plotted against the frequency. The inset illustrates the sandwich structure used for the capacitance and leakage current measurements (Drawn by C.W.). (**f**) Leakage current of the GO/chitosan composite electrolyte film.

Figure 1a shows the chemical structures of GO sheet and chitosan. They were dispersed in water solution with 0.5 mg/mL for GO sheets and 2.0 wt% for chitosan. The GO and chitosan solutions were then mixed at a weight ratio of 1:1 at room temperature and stored in a glass bottle for the subsequent use (Fig. 1b). To make GO/chitosan composite film, the as-obtained solution containing GO sheets and chitosan was spin-coated on substrates and dried in air naturally. To investigate the ionic conducting characteristics of the GO/chitosan composite film, which was critical for realizing low-voltage operation of our device, Nyquist plots, specific capacitance and leakage current were measured by using a sandwich IZO/hybrid/ITO structure.

**Figure 2:**
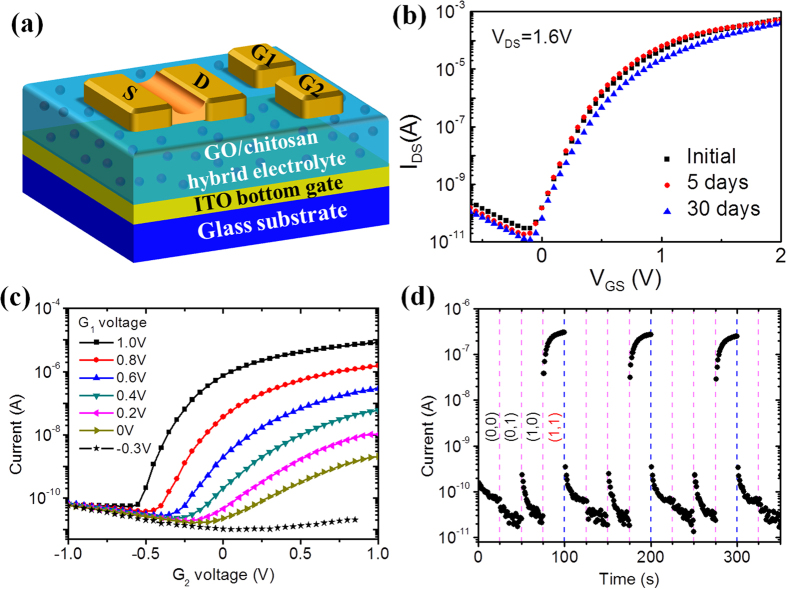
AND logic demonstrated in a two-gate IZO-based EDL transistor using GO/chitosan composite as gate dielectrics. (**a**) Schematic of IZO-based EDL transistor using inorganic/organic GO/chitosan composite film as gate dielectric (Drawn by C.W.). G_1_ and G_2_ are lateral gates. (**b**) Aging tests of the transfer curve measured by sweeping the bottom gate voltage. (**c**) The transfer curves measured by sweeping the voltage on lateral gate G_2_ can be tuned by G_1_. (**d**) AND logic operation using two lateral gates as inputs. During measurements, 0 and 1 denote the voltages of −1.0 and 1.0 V, respectively.

Figure 1c shows Nyquist plots of the GO/chitosan composite film, which depicts real/imaginary parts of the impedance. During measurements, an alternating-current (AC) potential was applied on the GO/chitosan film. The plots displayed an inclined spur in the low-frequency region and a partial semicircle in the high-frequency region. These curves are fingerprints of ion conductors contacted by blocking electrodes, with the semicircle corresponding to the bulk ionic impedance and the inclined spur to electroactive species at electrodes^51^,^52^. The curves can be fitted by a simple equivalent circuit that consists of a capacitor in series with both a resistor and a capacitor, which correspond to the film/electrode interface capacitance, the film bulk resistance and the film bulk capacitance, respectively. This equivalent circuit has been used to model ionic conductor accurately by accounting for the bulk impedance and capacitive effect at contacts^53^,^54^,^55^. This model could be used for our GO/chitosan composite electrolyte film. The impedance real value (*R*) is obtained with the impedance imaginary value is equal to zero. The conductivity (*σ*) can be obtained from the equation *σ = D/(R-R*_*0*_*)A*, where *D, A* and *R*_*0*_ are the thickness of the composite electrolyte film, the surface area of electrode, and the resistance of the electrodes, respectively^53^,^54^,^55^. In our system, *D* is ~10 μm, *A* is ~1.5 × 10^–3^ cm^2^ and *R*_*0*_ is measured to be ~30 Ω. For GO/chitosan composite, R is about 520 Ω, thus an effective proton conductivity of ~13.6 × 10^–4^ S/cm is calculated. For comparison, the Nyquist plot of pure chitosan is shown by red dots in Fig. 2c and an effective proton conductivity of ~7.56 × 10^–4^ S/cm can be realized.

Figure 1d shows the cross-sectional scanning electron microscope (SEM) image the fabricated GO/chitosan composite electrolyte film. The thickness was measured to be ~10 μm. Figure 1e shows the capacitance of the GO/chitosan composite electrolyte film plotted against the frequency measured using an IZO/electrolyte/ITO sandwiched structure. With increasing the frequency in logarithmic coordinate, the capacitance decreases nearly linearly. After about 200 Hz, the capacitance decreases much quicker. The specific capacitance is about 3.2 μF/cm^2^ at 1.0 Hz. This value is comparable with CVD-deposited porous silica dielectrics^21^ and about two orders of magnitude larger than conventional intrinsic silica with a thickness of hundreds of nanometers. The frequency-dependent specific capacitance is typically found in ion-based electrolyte materials, suggesting a strong contribution of mobile ions with slow relaxation. Figure 1f shows the leakage current curves of the GO/chitosan composite electrolyte film. A low leakage current is obtained in the operation voltage range from −2.0 to 2.0 V and in this voltage range the maximum leakage current of the composite electrolyte film is about 3 nA at −2.0 V.

After depositing the GO/chitosan composite electrolyte film on ITO-coated PET substrates, the devices were fabricated and the details are in the Method section. The final IZO-based EDL transistor is schematically illustrated in Fig. 2a. The device has a self-assembled IZO channel layer between the source and drain electrodes. The two lateral gate electrodes can tune the conductance of the channel by lateral EDL coupling. Electrical properties of the EDL transistor associate closely with interfacial charging status between the gate dielectric and the semiconducting oxide channel^56^,^57^,^58^. Firstly, we measured the transfer (I_DS_−V_GS_) curve of the device by using the bottom gate (Fig. 2b). The source-drain voltage V_DS_ is 1.6 V. The gate voltage V_GS_ is sweeping from −1.0 to 2.0 V at a rate of 0.05 V/s. With increasing V_GS_, the channel current I_DS_ decreases gradually at first and then increases rapidly after surpassing the turn point where V_GS_ is about −0.1 V. Low-voltage and enhancement mode operation is clearly realized. Moreover, we performed aging test on our devices by storing them in air condition without applying bias stress. The transfer curves of our device are stable after 5 and 30 days aging, as shown by the red and blue lines in Fig. 2b, respectively.

The modulation of carrier density through carrier accumulation or depletion using ion gels and electrolytes as gate dielectrics has been used to effectively tune the carrier density of the channel layers for the realization of novel properties such as interfacial superconductivity and insulator-to-metal transition^59^,^60^,^61^. Organic and oxide semiconductors which can form good interface with electrolyte dielectrics are usually used as the channel materials of these devices^59^,^60^,^61^,^62^,^63^,^64^. In our system, there are protons in the composite electrolyte. Similar to operation mechanism of EDL transistors reported by others^59^,^60^,^61^,^62^,^63^,^64^, the migration of protons towards and away from the electrolyte/channel interface, and the corresponding electron concentration modulation in the channel due to the EDL effect, is expected to be the main working mechanism of our device. Also, to exclude water electrolysis as a possible mechanism of our device is discussed. In water electrolysis, the reduction and oxidation reaction mainly occur at the electrodes which are usually made from inert metal materials such as platinum, stainless steel or iridium. Moreover, the reaction of water electrolysis has a standard potential of −1.23 V, meaning it requires a potential difference of 1.23 V to split water. In contrast, the adsorption of water molecule is in GO/chitosan composite electrolyte in our device. In addition, conductive oxide materials IZO and ITO are used as the electrodes and the operation voltage of our device is lower than the potential difference required for water electrolysis. We thus think water electrolysis mechanism is not important in our system.

In addition, in our EDL transistors, the lateral in-plane gate voltage can also tune the carrier concentration in the channel layer due to proton migration in the composite electrolyte. Our measurements show that transfer curves of one lateral gate G_2_ can be tuned by the voltage applied on another lateral gate G_1_ and the results are shown in Fig. 2c. During the measurements, V_DS_ is 0.5 V and the voltage on G_2_ is changed from −1.0 to 1.0 V. The results indicate that the transfer curve can be effectively tuned by the voltage on G_1_. As the voltage on G_1_ increases to 1.0 V, the current across the channel can be modulated by about five orders of magnitude by using G_2_; in contrast, for a negative voltage of −0.3 V applied on G_1_, the transistor is almost depressed. Such a characteristic can be understood by considering the change of proton distribution in the dielectric film below the IZO channel layer. When the voltage on G_1_ is negative, protons in the dielectric film will be gathered around lateral gate G_1_. There are not enough protons to be used by G_2_ to tune the channel layer. In contrast, when the gate voltage on G_1_ is positive, the protons will be pushed away and gathered below the channel layer. These protons can be used to tune the channel layer by G_2_.

Since the channel layer can be tuned by lateral gate voltage, the EDL transistor with multiple lateral gates can be used to realize spike logic AND operation (Fig. 2d). In a typical test, −1.0 and 1.0 V voltages are regarded as binary 1 and 0, respectively. When the voltage −1.0 V is applied on G_1_ or on both G_1_ and G_2_, the current across the channel layer is at a low level. It means that in the cases of (0, 0), (0, 1) and (1, 0), the output is 0. In contrast, when the voltage 1.0 V is applied on G_1_ and G_2_, the output is 1. Logic AND operation is successfully realized in our system. It should be noted that the logic AND operation is realized in a single device here, which is different from conventional Si-based electronics that are extensively used currently.

**Figure 3:**
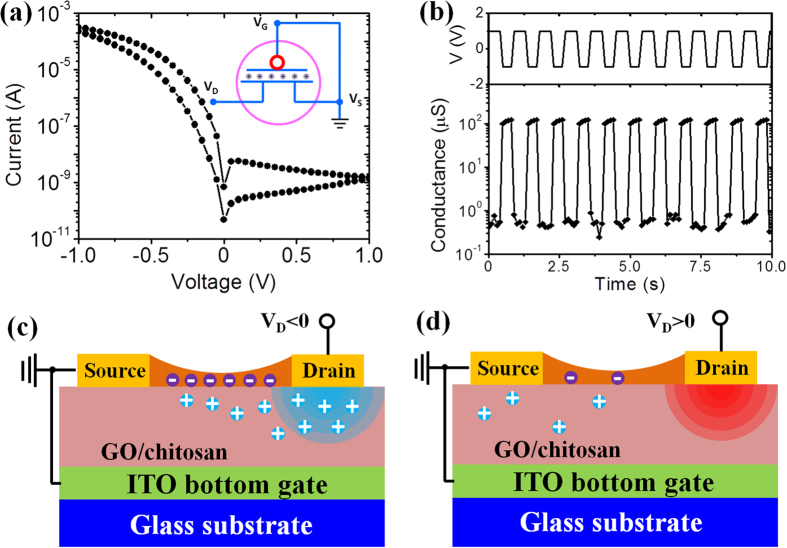
Two-terminal operation of the device. **(a**) Current-voltage curve of the device. Absolute value of the current is used. Inset is the schematic illustration of two-terminal operation. (**b**) Switching characteristics of the device. The channel conductance is recorded (the bottom panel) simultaneously when the drain voltage is switching between 1.0 and −1.0 V (the top panel). (**c**), (**d**) Schematics of the working mechanism (Drawn by C.W.). When the drain voltage is negative, protons will be attracted below the channel layer, inducing free electrons in the channel layer due to the EDL effect; however, when the drain voltage is positive, protons will be pushed away from the channel, and thus there are fewer electrons in the channel layer.

Based on the above results, it could be found out that our devices operated on the basis of voltage-induced proton migration in the composite electrolyte dielectric, which could tune the proton concentration at the dielectric/channel interface. The channel conductance can thus be tuned by EDL coupling effect. If the source and bottom gate are grounded, the drain voltage is expected to have dual functions of simultaneously measuring channel conductance and tuning free electron concentration of the channel layer. Two-terminal operation layout of our device is schematically shown in the inset of Fig. 3a. The corresponding current-voltage curve is shown in Fig. 3a. In order to show the current-voltage characteristics in logarithmic scale, absolute value of the current is used. With changing the voltage, the current decreased rapidly at first when the voltage was biased at the negative side and was at a low level for positive voltage. In this voltage range, the current across the channel layer could be changed by about five orders of magnitude, indicating that the conductance of the channel could be significantly tuned by the drain voltage when the source and gate were grounded together. Since the drain voltage could tune the channel conductance in the two-terminal condition, the device could operate as a switch. Figure 3b shows the characteristics of the device by switching the voltage between 1.0 and −1.0 V. The current across the channel is recorded simultaneously. The ON-state conductance of the device is ~100 μS at a drain voltage of −1.0 V. The OFF-state conductance of the device is below 1 μS at 1.0 V.

Accounting for mobile protons in the GO/chitosan composite electrolyte film, a proposed working mechanism is given. When the drain voltage is negative (Fig. 3c), protons will be attracted to the region below the IZO channel. An EDL is formed at the interfacial regime, thus a higher concentration of free electrons can be induced in the channel due to the EDL effect. As a result, the channel conductance is high. However, in contrast, when the drain voltage is positive (Fig. 3d), protons will be pushed away and there are fewer electrons in the channel layer. Accordingly, the channel conductance is at a low level. Due to the strong EDL capacitive coupling, transistors gated by such electrolytes can operate at low voltages. Our GO/chitosan composite electrolyte film shows a high capacitance of ~3.2 μF/cm^2^ at 1.0 Hz. For comparison, normal SiO_2_ and HfO_2_ with the thickness of 100 nm have capacitance of 0.035 and 0.22 μF/cm^2 ^65 respectively, much lower than our GO/chitosan composite electrolyte gate dielectric. Therefore, our device can operate at a low voltage range of −2.0 to 2.0 V. In the case of Fig. 2b, the gate voltage V_GS_ is applied on the bottom ITO gate electrode; when V_GS_ is positive, protons will accumulate at the dielectric/channel interface and induce electrons in the channel layer, so the IZO channel current (between IZO source/drain electrode) is high. In the case of Fig. 3a, the voltage is applied on the IZO drain electrode and the IZO source electrode and bottom ITO gate electrode are grounded. When the drain voltage is negative, protons will migrate to the area near interface of electrolyte/IZO channel, and the free electron concentration in the IZO channel is high, and the measured channel current is large. When the drain voltage is positive, protons will migrate to the area near the bottom ITO gate electrode, and the free electron concentration in the IZO channel is low, and the measured channel current is low.

Previously, two kinds of artificial electronic/ionic devices are mainly used to emulate synaptic behaviors^24^,^25^,^26^,^27^,^28^,^29^,^30^,^31^. One is resistor-based two-terminal devices and another is three-terminal electrolyte-gated EDL transistors. Furthermore, some organic electronic devices have been found to have functional interfaces with neural activity^66^,^67^,^68^,^69^. Here we will present that our EDL transistors can also function as a two-terminal device to emulate synapses. Because our device is working on the basis of proton migration in the GO/chitosan composite electrolyte and the relaxation process of protons is relatively slow, synaptic-like short-term plasticity behaviors can be realized. When an excitation voltage spike is applied on the drain terminal, due to the slow relaxation of protons, proton distribution at the dielectric/channel interface will be changed gradually. When the voltage spike is finished, protons will return to the equilibrium state slowly. If subsequent voltage spikes are applied on the drain before the equilibrium state is reached, proton distribution is synergistic modulated by these voltage spikes. In other words, subsequent voltage spikes applied on the drain can be coupled temporally. This coupling can be transduced to electron concentration in the channel due to the EDL effect and reflected by channel conductance, resulting in the realization of short-term synaptic plasticity including memory and filtering functions in our system.

**Figure 4:**
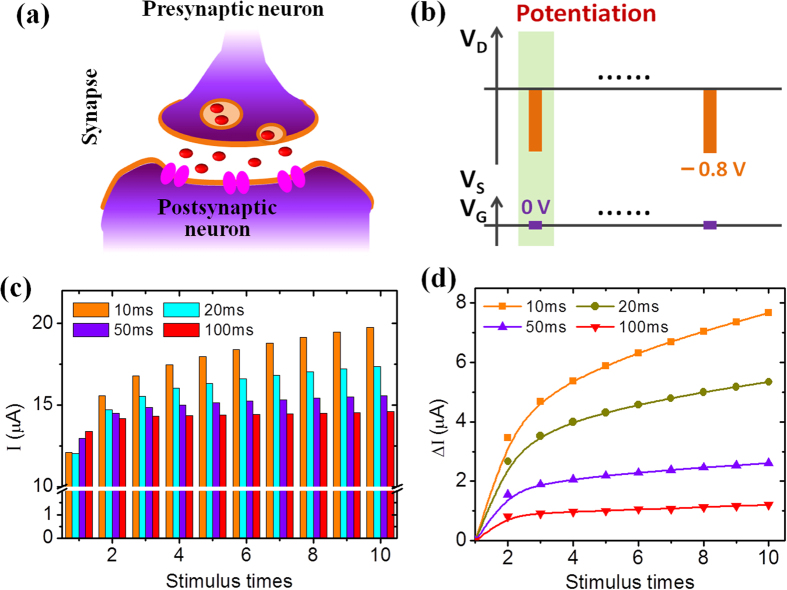
Synaptic behavior emulation and the dependence of the transition efficiency on stimulation rate. (**a**) Schematic of spatial excitation in a synapse which is utilized by neurons for cell-cell interaction (Drawn by C.W.). (**b**) Voltage pulses with the height of −0.8 V and duration of 10 ms are used to fire the channel. (**c**) The channel current recorded after each stimulation voltage pulse at different pulse interval conditions. (**d**) Current increase ∆I after every stimulus plotted against the pulse number for different pulse interval conditions.

Firstly, the response of the device to different stimulation rates is investigated at the two-terminal mode. Figure 4a shows the schematic of a synapse. Whenever a stimulus triggers an action potential, a neuron transmits the signal to the next through synapses. First, an action potential reaches at the presynaptic membrane (axon terminal), where it contains neurotransmitters packed in synaptic vesicles. The action potential triggers the release of neurotransmitters to the synapse as synaptic vesicles diffuse into the membrane. Neurotransmitters reach receptors located on the postsynaptic membrane (dendrite terminal). This triggers an action potential at the postsynaptic membrane which is transmitted to the next neuron. In our device, presynaptic inputs are voltage pulse applied on the drain terminal and the channel conductance is regarded as the synaptic weight.

Figure 4b shows the measurement method of stimulation-rate dependent synaptic response where voltage pulses with the height of −0.8 V and the duration of 10 ms are used to fire the channel and the postsynaptic currents are recorded simultaneously. The voltage pulses can modulate free carrier concentration in the channel according to proton-mediated EDL coupling effect. The time interval Δt between voltage pulses is changed and the results are shown in Fig. 4c. A clear dependence of conductance enhancement on the stimulation rate is observed, with a high stimulation rate being most effective and low stimulation rate being least effective. For Δt = 10 ms, the current across the channel increases rapidly with the number of stimulation pulses. With increasing Δt, the channel current increases much slowly. To qualify the conductance enhancement, the current increase ΔI is defined as the difference between the measured current and the current of stimulation 1. Figure 4d shows the current increase ΔI after every voltage stimulus as a function of the pulse number at different conditions. At shorter time interval, the value increases much quicker. This phenomenon is expected to relate to the proton relaxation process in the GO/chitosan composite electrolyte. Upon negative voltage excitation, the protons in the electrolyte dielectric will be pushed towards the channel layer; in contrast, when the pulse is off, some protons will move away from the interfacial region gradually. At a shorter pulse interval, more protons will be left below the channel at the same stimulation number and free electron density in the channel is higher. Accordingly, the channel current increases gradually with the stimulations and the current increase is higher at shorter pulse interval.

In neuroscience, synapse refers to the junctions between vastly interconnected networks of neurons and allows pre-synaptic neuron to pass signals (action potential in the form of spikes) to a post-synaptic neuron or cell with the ability of long-term or short-term changing of its efficacy (synaptic weight)^70^,^71^. The change by activity history could be enhancement or depression and such activity-dependent synaptic plasticity is believed to be in charge of memory and learning. Synapse/neuron can also act as dynamic filter for information processing depending on signal frequency^71^. Multiple biological signals and mechanisms can regulate the efficacy of synaptic transmission, thereby providing rich combinatorial possibilities for modifying neural communication. Synaptic efficacy can increase/decrease a lot within milliseconds after the onset of specific temporal patterns of the activity. Short-term synaptic depression can be functioned as low-pass filtering while short-term synaptic facilitation can be functioned as high-pass filtering.

**Figure 5:**
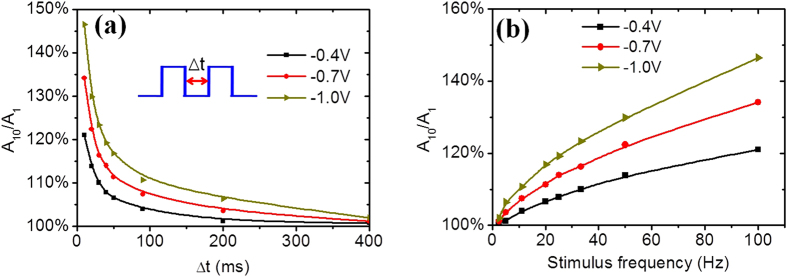
Synaptic-like high-pass filtering characteristics. A series of presynaptic spike trains with controlled pulse and frequency are applied on the drain and the current across the channel is recorded. The amplitude ratio between the last and the first spike is defined as the amplitude gain, which is plotted against the spike interval (**a**) and the stimulus frequency (**b**), respectively, for different pulse heights.

To evaluate the filtering characteristics of the device at two-terminal operation mode, we test excitatory postsynaptic currents (EPSCs) of the device when a series of presynaptic spike trains with different frequencies are applied on the drain. Each stimulus train contains 10 voltage spikes. At a higher frequency, the EPSC increases more intensively with successive spike stimulus. With decreasing the frequency of stimulus trains, the peak value of spikes decrease gradually. To describe the filtering characteristics of our system, the amplitude ratio between the last and the first spike is defined as the amplitude gain, which is plotted as a function of the pulse interval Δt (Fig. 5a). With increasing the spike interval, the amplitude gain decreases nearly exponentially. In addition, the amplitude gain can also be tuned by the pulse height. With increasing the pulse height, the amplitude gain increases. The amplitude gain can be plotted against the frequency of presynaptic spikes (Fig. 5b). High-pass filtering characteristics are realized in our system. With increasing stimulus frequency, the amplitude gain increases nearly linearly, indicating a stronger coupling between spikes at a higher stimulus frequency. This behavior of our device is similar to the high-pass filtering function of the biological neuron. This filtering function, which can augment the synaptic response to high-frequency inputs and diminish the impact of low-frequency inputs, is important for neural computation.

## Conclusions

In summary, it was demonstrated that solution-processed GO/chitosan composite film was an excellent proton conducting electrolyte with a high capacitance of ~3.2 μF/cm^2^ at 1.0 Hz. Self-assembled multi-gate IZO-based EDL transistors gated by such composite electrolyte were fabricated. We found that the bottom and lateral gate voltages as well as the drain voltage could be used to tune the transistor channel conductance, resulting in the realization of diverse functions of our device. First, dual-gate AND logic operation was realized in a single device. Second, two-terminal operation was realized because the drain voltage can tune free electron concentration of the IZO channel layer. Third, our oxide-based EDL transistors could operate as a two-terminal artificial synapse to emulate the synaptic behaviors for neuromorphic applications. Overall, our experimental results present an interesting new-concept device application of GO/chitosan composite electrolyte film.

## Methods

### Preparation of GO/Chitosan Composite Electrolyte Films

GO sheets were dispersed in water at 0.5 mg/mL. Chitosan powders (>99.5%, Aldrich) were dissolved in acetic acid solution to form 2.0 wt% chitosan solution. GO solution was then added gradually into chitosan solution under magnetic stirring and the two solutions were mixed at a volume ratio of 1:1. The solution composed of GO and chitosan was then spin-coated at a speed of 500 rpm on ITO-coated PET substrate and then dried in air naturally to form GO/chitosan composite film.

### Fabrication of the Devices

The channel layer and electrodes (source, drain and lateral gate electrodes; dimension: L × W = 150 μm × 1000 μm) of the devices are composed of indium-zinc-oxide (IZO) film deposited by radio-frequency (RF) magnetron sputtering at room temperature. Neither any heating treatment nor post-annealing treatment was adopted. The weight ratio of In_2_O_3_ and ZnO in the IZO target is 90%:10%. During the sputtering process, the Ar flow rate, the pressure and the RF power density were 30 sccm, 0.5 Pa and 5.3 W/cm^2^, respectively. A nickel shadow mask was used to pattern the IZO film. The spacing between the source and drain electrodes on the mask is 80 μm. The mask was placed at a distance of about 30 μm above the GO/chitosan hybrid membrane. During the sputtering process, sputtered particles can bypass the nickel shadow mask to form a thin IZO channel layer with a thickness of ≈20 nm between the patterned IZO source/drain electrodes. The final IZO-based EDL transistors using GO/chitosan composite film as gate dielectric are schematically shown in Fig. 2a. The length and width of the self-assembled IZO channel are 80 and 1000 μm, respectively. The thickness of the source, drain and lateral gate electrodes is about 100 nm.

### Electrical Measurement of the Transistors

The electrical measurements were carried out on an EverBeing probe station equipped with Keithley 2636B Source/Measurement Units at room temperature. For all of the measurements, the relative humidity level was controlled at 50%.

## Additional Information

**How to cite this article**: Feng, P. *et al*. Proton Conducting Graphene Oxide/Chitosan Composite Electrolytes as Gate Dielectrics for New-Concept Devices. *Sci. Rep.* **6**, 34065; doi: 10.1038/srep34065 (2016).
